# Therapeutic Landscape of Rosacea: From Clinical Trials to Future Directions

**DOI:** 10.1111/jocd.70182

**Published:** 2025-04-30

**Authors:** Sitong Li, Jiacheng Lin, Jiaqi Li, Xiaohui Mo, Qiang Ju

**Affiliations:** ^1^ Department of Dermatology, Renji Hospital Shanghai Jiao Tong University School of Medicine Shanghai People's Republic of China

**Keywords:** clinical trial, rosacea, treatment

To the Editor,

Rosacea is a chronic inflammatory skin condition characterized by facial flushing, persistent erythema, papules, pustules, and telangiectasia that impair patients' quality of life [1]. Despite its prevalence, the pathogenesis remains poorly understood, and current treatments often show limited efficacy [2]. This study analyzes rosacea‐related clinical trials to identify research gaps and therapeutic directions. We searched ClinicalTrials.gov on September 28, 2024, identifying 186 eligible interventional studies. We evaluated trial phases, start years, and primary purposes, focusing on 138 drug‐related and 26 non‐drug trials.

Between 2002 and 2024, 186 rosacea‐related trials were registered across all phases (Figure 1). Of these, 42 trials did not specify a phase, likely reflecting non‐traditional designs or unique interventions. Among 144 trials with defined phases, Phase 2 trials were most frequent (*n* = 46), followed by Phase 3 (*n* = 41). From 2002 to 2010, trial numbers remained relatively low during this exploratory stage. The period from 2011 to 2017 marked peak activity, particularly in Phase 2 and 3 trials, indicating the advancement of many therapies into mid‐ and late‐stage clinical validation. Although registrations decreased from 2018 to 2024, research interest remains substantial. Of the 186 trials, 164 focused on treatment, while 22 addressed non‐treatment areas, including basic science(*n* = 6), diagnostics(*n* = 2), supportive care(*n* = 5) and other intervention(*n* = 9). Among treatment‐focused trials, non‐drug interventions (*n* = 26) included dietary supplements (*n* = 2), skincare products (*n* = 6), and physical therapies (*n* = 18). Drug‐based trials were dominated by topical treatments (*n* = 109), followed by oral (*n* = 23), injectable (*n* = 9), and ophthalmic (*n* = 4) routes.

**FIGURE 1:**
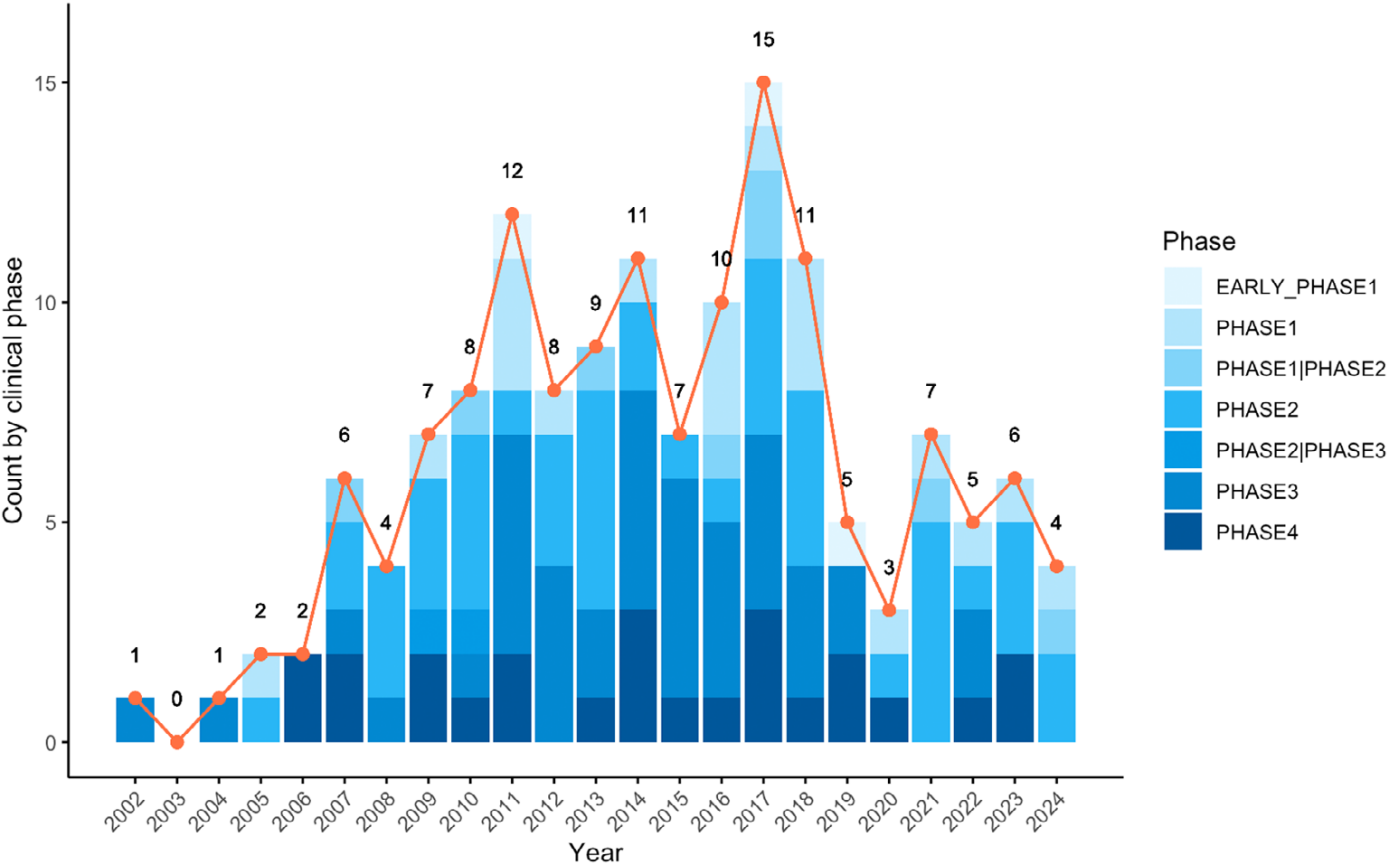
The distribution of clinical trials across different phases and years.

Our analysis of drug categories and therapeutic targets reflects the ongoing effort to address rosacea's complex pathophysiology through diversified treatment strategies (Figure 2). Antimicrobial agents were most common (36.55%), followed by vasoconstrictors (20.69%) and anti‐inflammatory drugs (14.48%). While traditional antimicrobial and anti‐inflammatory therapies remain central, the increasing use of vasoconstrictors reflects growing recognition of vascular regulation as a critical therapeutic target. Since 2007, vascular abnormalities have attracted attention for their role in persistent erythema and flushing. VEGF polymorphisms may contribute to pathogenesis by promoting vascular dysfunction and inflammatory responses [3]. Novel targets, such as IL‐17 and TYK2 inhibitors, suggest innovative pathways in immune regulation [4], while TRPV1 antagonists highlight the potential of neural pathway modulation [5]. Since 2011, research has increasingly focused on immune and neural regulation, particularly examining the relationship between Demodex mites and immune dysfunction. The expanding use of ivermectin demonstrates its dual function in controlling mite infestation and modulating immune responses [6]. Physical therapies, including PDL and IPL, have been incorporated to improve vascular symptom control. The limitations of single‐target therapies have driven the exploration of multi‐target combinations. Beyond optimizing existing drugs through novel formulations (foams and powders), several innovative therapies, such as ATR‐04 (bacterial substitute) and deucravacitinib (TYK2 inhibitor), have entered clinical trials.

**FIGURE 2:**
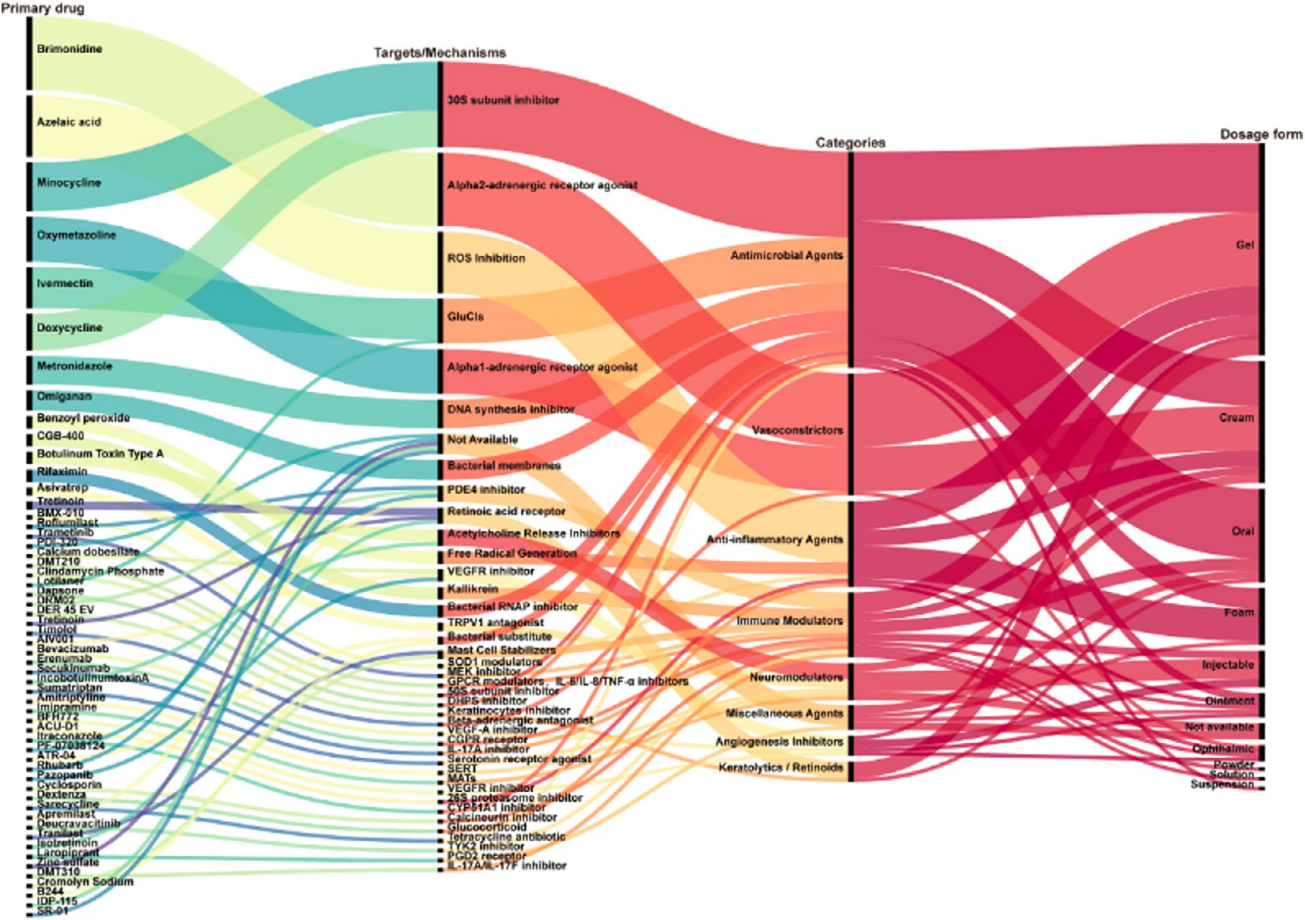
A Sankey diagram illustrating the relationships between primary drugs, their mechanisms of action, drug categories, and dosage forms. GluCls, Glutamate Chloride Channels; PDE4, Phosphodiesterase 4; VEGFR, Vascular Endothelial Growth Factor Receptor; VEGF‐A, Vascular Endothelial Growth Factor‐A; RNAP, RNA Polymerase; TRPV1, Transient Receptor Potential Vanilloid 1; SOD1, Superoxide Dismutase 1; MEK, Mitogen‐Activated Protein Kinase Kinase; GPCR, G‐Protein‐Coupled Receptor; IL‐6, Interleukin 6; IL‐8, Interleukin 8; TNF‐α, Tumor Necrosis Factor Alpha; DHPS, Dihydropteroate Synthase; CGPR, Calcitonin Gene‐Related Peptide Receptor; IL‐17A, Interleukin 17A; IL‐17F, Interleukin 17F; SERT, Serotonin Transporter; MATs, Monoamine Transporters; TYK2, Tyrosine Kinase 2.

This study has limitations due to restricted information availability in ClinicalTrials.gov. We supplemented our analysis with data from DrugBank and Synapse to better understand drug mechanisms and targets. Our findings reveal key trends: (1) sustained research interest in rosacea, (2) diversified therapeutic strategies encompassing antimicrobial, immune‐modulatory, and neuro‐vascular‐regulatory approaches, (3) continued focus on topical formulations, and (4) emergence of new targets and innovative formulations. Although progress has been made, unmet clinical needs persist, emphasizing the importance of personalized treatment strategies and long‐term efficacy evaluations.

## Disclosure

The authors have nothing to report.

## Conflicts of Interest

The authors declare no conflicts of interest.

## Data Availability

The authors have nothing to report.
